# The Close Encounter Between Alpha-Synuclein and Mitochondria

**DOI:** 10.3389/fnins.2018.00388

**Published:** 2018-06-07

**Authors:** Mattia Vicario, Domenico Cieri, Marisa Brini, Tito Calì

**Affiliations:** ^1^Department of Biomedical Sciences, University of Padova, Padova, Italy; ^2^Department of Biology, University of Padova, Padova, Italy; ^3^Padova Neuroscience Center, University of Padova, Padova, Italy

**Keywords:** alpha-synuclein, mitochondria, Parkinson disease, neurodegeneration, bioenergetics

## Abstract

The presynaptic protein alpha-synuclein (α-syn) is unequivocally linked to the development of Parkinson’s disease (PD). Not only it is the major component of amyloid fibrils found in Lewy bodies but mutations and duplication/triplication in its gene are responsible for the onset of familial autosomal dominant forms of PD. Nevertheless, the precise mechanisms leading to neuronal degeneration are not fully understood. Several lines of evidence suggest that impaired autophagy clearance and mitochondrial dysfunctions such as bioenergetics and calcium handling defects and alteration in mitochondrial morphology might play a pivotal role in the etiology and progression of PD, and indicate the intriguing possibility that α-syn could be involved in the control of mitochondrial function both in physiological and pathological conditions. In favor of this, it has been shown that a fraction of cellular α-syn can selectively localize to mitochondrial sub-compartments upon specific stimuli, highlighting possible novel routes for α-syn action. A plethora of mitochondrial processes, including cytochrome c release, calcium homeostasis, control of mitochondrial membrane potential and ATP production, is directly influenced by α-syn. Eventually, α-syn localization within mitochondria may also account for its aggregation state, making the α-syn/mitochondria intimate relationship a potential key for the understanding of PD pathogenesis. Here, we will deeply survey the recent literature in the field by focusing our attention on the processes directly controlled by α-syn within mitochondrial sub-compartments and its potential partners providing possible hints for future therapeutic targets.

## Introduction

Parkinson’s disease (PD) is the second most common neurodegenerative disease, affecting more than 50000 people each year only in the US^1^^,^^2^ . Worldwide, more than 10 million people are estimated to suffer from PD. Clinically, the selective loss of dopaminergic neurons in the substantia nigra pars compacta leads to motor dysfunction and the appearance of bradykinesia, resting tremor, rigidity and postural instability. Although some approved drugs alleviate the symptoms of PD, the disease is still incurable.

**FIGURE 1:**
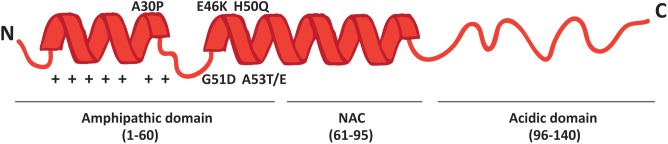
Schematic representation of α-syn structure. The protein forms two alpha-helices when interacting with lipids and is composed of three distinct domains: the N-terminal amphipathic region, the non-amyloidogenic component (NAC) and the C-terminal acidic domain. The N-terminal domain drives α-syn to mitochondria thanks to the presence of seven positively charged lysine residues (indicated as +) and contains all the PD-related mutations.

More than 90% of PD cases are sporadic. A small percentage, however, is due to mutations in specific genes. *SNCA* was the first gene to be associated to familial cases of PD (Polymeropoulos et al., 1997) and it encodes alpha-synuclein (α-syn), a small 14 kDa protein that was also found to accumulate in the Lewy bodies, the proteinaceous structures that mark the histopathology of PD (Spillantini et al., 1998). The 140 amino acids sequence of α-syn can be divided in three major regions (**Figure 1**): the amphipathic N-terminus, the hydrophobic central non-amyloid-beta component (NAC) and the acidic C-terminal domain.

The N-terminal domain is enriched in lysines and is characterized by the presence of 7 repeats of 11 residues each among which the KTKEGV motif is the core consensus sequence. The pathological mutations (A30P, A53E/T, E46K, G51D and H50Q) cluster in this domain, which is likely involved in the interaction with membranes. Indeed, the A30P mutation disrupts the interaction of α-syn with lipid rafts and causes the redistribution of α-syn in the axon (Jensen et al., 1998; Fortin et al., 2004). The NAC region was originally discovered in amyloid from the cortex of Alzheimer’s disease patients (Ueda et al., 1993) and is essential for the aggregation of α-syn molecules, since deletions within this region abrogate the assembly of α-syn into filaments both *in vitro* and in intact cells (Giasson et al., 2001; Luk et al., 2009). The C-terminal tail displays many charged residues, lacks secondary structure bias and contains the pathologically relevant phosphorylation sites cluster.

The expression of α-syn is limited to the subphylum of vertebrates. It is highly expressed in both the central and peripheral nervous system and it is particularly enriched in the nerve terminal (Maroteaux et al., 1988; Yang et al., 2010; Vivacqua et al., 2011); at lower levels, it is also present in other tissues like hearth and muscles^3^.

The function of α-syn within the cell is still not completely clear, but its localization at presynaptic terminal reflects a role in synaptic transmission. α-syn co-localizes with synaptic vesicles and plays a role in the fast kinetics of synaptic vesicle endocytosis, indeed, this process is impaired in αβγ-syn^-/-^ neurons and rescued by α-syn reintroduction (Lee et al., 2008; Zhang et al., 2008; Vargas et al., 2014). The number of presynaptic vesicles was reduced in cultured neurons from α-syn knockout mice (KO) as well as the size of synaptic puncta in αβγ-synuclein triple KO, (Cabin et al., 2002; Greten-Harrison et al., 2010). At the synapses, α-syn promoted the assembly of the SNARE complex by interacting with synaptobrevin 2 (VAMP2) and phospholipids through its C- and N- terminus, respectively (Burre et al., 2010).

In addition to its role at synapses, α-syn plays a role in the nucleus. Its name, indeed, reflects a nuclear localization (Maroteaux et al., 1988; Mori et al., 2002; Goncalves and Outeiro, 2013). Within the nucleus, α-syn affects the expression of several genes, including downregulation of major genes involved in DNA repair leading to increased levels of phosphorylated p53 and reduced levels of acetylated histone 3 (Paiva et al., 2017) and impaired neurogenesis by modulating Notch1 expression (Desplats et al., 2012). Interestingly, upon oxidative stress induction, α-syn nuclear localization increases and the protein binds to the promoter of several genes including PGC-1alpha, a transcription co-activator involved in metabolism and mitochondrial biogenesis (Siddiqui et al., 2012).

Either gene multiplications or point mutations in the *SNCA* gene are at the basis of familial dominantly inherited PD and result in the accumulation and aggregation of the protein in Lewy bodies within the brain (Conway et al., 1998; Pandey et al., 2006; Ghosh et al., 2013; Kara et al., 2014). The finding that gene multiplication is linked to PD suggests that α-syn expression level must be kept under tight control: when it reaches a specific threshold, wild type (wt) α-syn is sufficient *per se* to trigger PD, possibly as a consequence of augmented aggregation propensity. The issue of α-syn aggregation and conformational structure is deeply investigated, but controversial reports have appeared so far. The soluble form is a natively unfolded monomer with no defined secondary structure (Weinreb et al., 1996), but the first 100 residues are predicted to be structured in an α-helical conformation (George et al., 1995; Wang et al., 2011) that can be induced upon binding to lipids (Davidson et al., 1998). The NAC domain is responsible for α-syn aggregation: long-range hydrophobic interactions between the C-terminus and this region, as well as electrostatic interactions between the C- and the N-termini, prevent α-syn aggregation. Increases in temperature or the binding of polyamine have been shown to perturb these interactions favoring aggregation (Bertoncini et al., 2005). Recently, an aggregation resistant tetrameric structure of α-syn was described (Bartels et al., 2011; Wang et al., 2011;Dettmer et al., 2013): evidence obtained in non-neuronal and neuronal cell cultures, brain tissue, living human cells and purified protein suggested that under non-denaturing conditions α-syn is a 58 kDa tetramer, whose formation is disturbed by the introduction of A30P, A53T, E46K, H50Q, and G51D PD-related mutations (Dettmer et al., 2015). However, a different study performed on human, rat and mouse brains as well as on cell lines and *Escherichia coli* demonstrated that α-syn exists rather as a disordered monomer (Fauvet et al., 2012). All together, these findings indicate that multimeric α-syn can co-exists together with the monomeric protein in a dynamic equilibrium within the cell and, when the fraction of the unfolded monomer increases, the α-syn-mediated pathology is triggered (Dettmer et al., 2015).

Regardless of the monomeric or oligomeric nature of α-syn, its aggregation represents a key pathological feature of PD. Both oligomers and fibrils have been reported to have toxic effects on the functionality and the survival of neuronal cells. A better understanding of the physiological function of α-syn within the cell as well as of the mechanisms involved in its aggregation and propagation in the brain is essential to find new therapeutic approaches for PD. A lot of effort has been put in the last 5 years in the understanding of both the process of α-syn aggregation and the pathways responsible for aggregates elimination. Recently, a new mitochondria-mediated pathway that degrades cytosolic proteins prone to aggregation has been described in yeast and human cells. It has been named MAGIC (mitochondria as guardian in cytosol) and consists in the import of aggregation-prone proteins within mitochondria, where they will be degraded by mitochondrial proteases (Ruan et al., 2017). This process highlights the role of mitochondria as guardian of cell integrity and points on the necessity to deeply understand α-syn action and distribution at mitochondrial level.

## Alpha-Synuclein Effects on Mitochondria

Mitochondria are crucial players in the pathogenesis of PD. It is surprising that many (if not all) of the genes responsible for the onset of familial forms of PD, indeed, converge on mitochondria (Cieri et al., 2017) and besides the clear role played by PINK1 and Parkin as key regulators of mitochondrial integrity (Narendra et al., 2008), the list of PD-related genes linked to mitochondria is longer and α-syn is not an exception.

In this paragraph, we will briefly summarize the effects induced by α-syn on mitochondria. Several reports show contrasting results on this topic. It must be stressed, however, that differences in α-syn-induced effects may be explained by the cell type used in the experiments as well as the transfection method used, that may strongly affect protein expression levels.

α–syn itself can affect the Ca^2+^ signaling within the mitochondria as it has been repeatedly reported to influence the Ca^2+^ exchange and the physical interaction between the ER and mitochondria, despite different groups reached different conclusions (Calì et al., 2012; Guardia-Laguarta et al., 2014; Paillusson et al., 2017). Interestingly, we have also shown that addition of exogenous recombinant α-syn to cell cultures leads to a dose dependent impairment of Ca^2+^ handling, with different doses showing different effects: increased mitochondrial Ca^2+^ transients were observed upon incubation with the 4 μM exogenous α-syn, whereas a reduction was measured upon treatment with 8 μM (Calì et al., 2012). Similarly, another group reported that α-syn-induced mitochondrial fragmentation is dependent on its expression levels: whereas the expression of low levels of wt, A53T or A30P α-syn induced mitochondrial fragmentation only in the case of A53T, higher expression of the protein resulted in mitochondrial fragmentation also in the case of wt α-syn (Pozo Devoto et al., 2017).

Dynamic processes such as mitochondrial fusion/fission and axonal transport are also influenced by α-syn. Mitochondrial fragmentation induced by overexpression of mutant (A53T, A30P, E46K) α-syn has been observed, although the effect of the wt was not consistently reported (Kamp et al., 2010; Nakamura et al., 2011; Gui et al., 2012; Guardia-Laguarta et al., 2014). The expression of α-syn in sensory neurons of living zebrafish embryos resulted in the fragmentation of mitochondria, occasionally leading to their swelling within the axon (O’Donnell et al., 2014). The mitochondrial pathology is also extended to their axonal transport and to the mtDNA: mitochondrial motility was indeed reduced by α-syn expression in SH-SY5Y cells and cultured neurons derived from human embryonic stem cells (Xie and Chung, 2012; Melo et al., 2017; Pozo Devoto et al., 2017) and α-syn transgenic mice display increased mitochondrial oxidative stress and DNA lesions (Bender et al., 2013).

How α-syn induces changes in mitochondrial morphology is still unclear. Some groups have shown a direct effect on the expression of mitochondria-shaping proteins (Gui et al., 2012; Xie and Chung, 2012; Menges et al., 2017), despite a general consensus has not been reached (Kamp et al., 2010; Nakamura et al., 2011; Guardia-Laguarta et al., 2014; Pozo Devoto et al., 2017). Alternatively, a direct effect induced by the binding of α-syn to the mitochondrial membrane has been proposed. Accordingly, *in vitro* studies have shown inhibition of membrane fusion by α-syn (Kamp et al., 2010), prompting to speculate that the binding of α-syn to the mitochondrial membrane may change the curvature of the outer mitochondrial membrane (OMM) and reduce its fusion with surrounding mitochondria. This hypothesis is supported by experiments showing that the selective targeting of wt and A53T α-syn, but not A30P, to the OMM induced mitochondrial fragmentation (Pozo Devoto et al., 2017).

Endogenous α-syn has also been shown to be required for the normal activity of the respiratory chain complexes (Ellis et al., 2005; Devi et al., 2008), we thus may hypothesize that its levels must be tightly regulated and kept under control. Deviations from the threshold levels inevitably affect cellular and mitochondrial functions and lead to the alterations hereby described. Accordingly, mutations may exert their pathogenicity because, by affecting aggregation propensity, they could contribute to compromise the availability of α-syn by sequestrating it in aggregates both within the cell and in specific cellular compartments or because mutated α-syn may quickly reach the dose that is required to induce mitochondrial and cellular dysfunction.

Indeed, impairment of the complex I function and increased production of reactive oxygen species (ROS) have been consistently observed both in the absence and in the presence of overexpressed α-syn and the expression of the A53T mutation exacerbated the defects (Devi et al., 2008; Loeb et al., 2010; Reeve et al., 2015). Mitochondrial membrane potential and ATP production were also affected upon exogenous administration of the recombinant wild-type and mutant α-syn (Banerjee et al., 2010).

## Alpha-Synuclein at the Mitochondria-Associated Membranes (MAMs)

Mitochondria-associated membranes (MAMs) are functionally specialized regions where the endoplasmic reticulum (ER) and mitochondria come in close proximity (Hayashi et al., 2009) and are in place to regulate several cellular processes including ER stress, unfolded protein response, cholesterol and phospholipid metabolism, mitochondrial division and dynamics, Ca^2+^ signaling and apoptosis (Rizzuto and Pozzan, 2006; Wozniak et al., 2006; Csordas and Hajnoczky, 2009; Simmen et al., 2010; Friedman et al., 2011).

Impaired functionality of the MAMs as well as changes in the number of contacts between ER and mitochondria have been recently associated to neurodegenerative diseases, including PD (Calì et al., 2012, 2013; Ottolini et al., 2013; Guardia-Laguarta et al., 2014; Rodriguez-Arribas et al., 2016), amyotrophic lateral sclerosis (ALS) (Stoica et al., 2014, 2016) and Alzheimer’s disease (Zampese et al., 2011; Area-Gomez et al., 2012; Hedskog et al., 2013; Schon and Area-Gomez, 2013; Area-Gomez and Schon, 2016, 2017). The presence of lipid raft-like domains at the MAMs and the intrinsic propensity of α-syn to interact with acidic phospholipids (Fortin et al., 2004), immediately suggested the possibility of a potential specific targeting and action of α-syn at these sites (**Table 1**).

**Table 1:** Effects of the different sub-mitochondrial α-syn localizations on mitochondrial dynamics.

α-syn localization	α-syn effects	
	*High expression*	*Low expression*
Mitochondria-associated membranes (MAMs)	↓ ER-mito contacts ↓ Mitochondrial Ca^2+^ uptake ↓ ATP synthesis ↑ Mitochondrial fragmentation	↑ ER-mito contacts ↑ Mitochondrial Ca^2+^ uptake ↓ ATP synthesis
(OMM)	↑ Mitochondrial fragmentation ↓ Mitochondrial size ↑ Mitochondrial nuclear clustering ↓ Mitochondrial respiration ↓ Mitochondrial proteins import ↑ ROS production ↑ mtDNA damage ↓ Mitochondrial membrane potential ↓ ATP synthesis
Inter-membrane space (IMS)	↑ mPTP opening ↓ Mitochondrial membrane potential ↑ ROS production ↑ Mitochondrial swelling ↑ Mitochondrial vacuolation ↓ Cristae number
Inner mitochondrial membrane (IMM)	↑ ROS production ↓ Complex I activity
Mitochondrial matrix	↑ ATP synthesis


One of the first evidence suggesting a potential role played by α-syn at the ER-mitochondria interface comes from the demonstration that overexpression of wt α-syn was able to sustain mitochondrial Ca^2+^ uptake by increasing the number of ER-mitochondria juxtapositions in SH-SY5Y and HeLa cells, while its down regulation impaired mitochondrial Ca^2+^ transfer and morphology (Calì et al., 2012). Of notice, in Hela cells the increase of α-syn levels over a certain threshold both by treatment with valproic acid (which acts on endogenous protein) and by incubation with exogenous recombinant TAT-α-syn leads to a reduction in mitochondrial Ca^2+^ uptake (Calì et al., 2012). This suggests the intriguing possibility that α-syn behavior at MAMs is dependent on its expression level, which is also known to affect its mitochondrial localization (see below) (Shavali et al., 2008; Liu et al., 2009).

**FIGURE 2:**
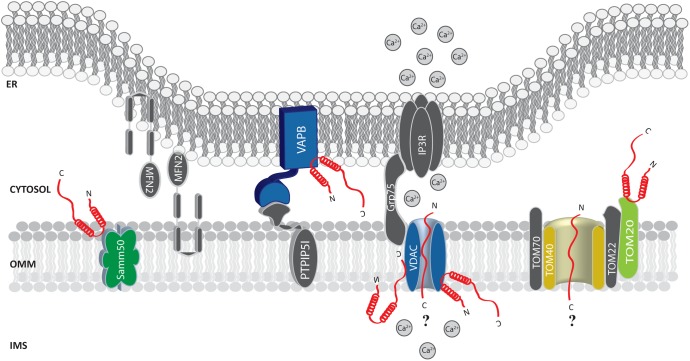
Alpha-syn at the mitochondria-associated membranes (MAMs) and the OMM. Picture shows α-syn binding partners at the MAMs and OMM. The protein’s interactors are highlighted in colors. The possible (?) mechanism/s of α-syn translocation inside mitochondria are also reported. A-syn is shown in red.

Soon after, α-syn was found at the MAMs (Guardia-Laguarta et al., 2014; Paillusson et al., 2017) and shown to interact with the ER vesicle-associated membrane protein-associated protein B (VAPB), thus perturbing its association with the protein tyrosine phosphatase–interacting protein 51 (PTPIP51) (**Figure 2**), and consequently their ER-mitochondria tethering function (De Vos et al., 2012). Thus, increased α-syn levels induced a decrease in the number of ER-mitochondria interactions in SH-SY5Y and iPS cell-derived dopaminergic neurons from a patient harboring *SNCA* triplication, followed by impaired inositol 1,4,5 triphosphate (IP_3_) receptor-mediated Ca^2+^ transfer to mitochondria and mitochondrial ATP production (Paillusson et al., 2017).

Additional evidence confirmed the pivotal role of α-syn at the MAMs, although different effects on the ER-mitochondria interface were reported for the wt and the mutant forms in different cell lines (Guardia-Laguarta et al., 2014). Whether this reflects cell line-specific features or is influenced by the different distribution within the cells of the wt and mutant proteins, remains to be elucidated.

Altogether, these data suggest that a portion of α-syn resides at the MAMs and influences some of their basic cellular activities. Altered α-syn expression, unbalanced equilibrium between the α-syn located in the MAMs, in the cytosol or mitochondria and the presence of PD-related mutations could contribute to the onset and the development of PD pathology by differentially interfering with MAMs functions (Guardia-Laguarta et al., 2015a,b).

## Mitochondrial Routes for Alpha-Synuclein

### Mitochondrial Localization of Alpha-Synuclein: General Evidence and Targeting Signals

The aforementioned data strongly imply that α-syn governs a plethora of mitochondrial processes. Whether these effects are regulated by α-syn directly or represent the culminating events of a signaling network arising from impairments in compartments other that mitochondria is still matter of investigation. Direct association of α-syn with mitochondria has been repeatedly and constantly observed in model cells and in different regions of the mouse brain (ventral midbrain, striatum and cortex) from α-syn transgenic mice (Subramaniam et al., 2014). Immunocytochemistry analysis (Parihar et al., 2008), immuno-gold electron microscopy (Parihar et al., 2008) and subcellular fractionation studies followed by western blot analysis (Subramaniam et al., 2014) revealed the presence of wt as well as PD-related A30P and A53T mutant α-syn in mitochondria. Interestingly, the presence of a fraction of α-syn residing in mitochondria was also found in dopaminergic neurons of substantia nigra from non-PD and PD subjects, but its accumulation was found only in mitochondria of PD patients (Devi and Anandatheerthavarada, 2010).

Albeit α-syn lacks a canonical mitochondrial targeting sequence, NMR studies and sequence alignments with cleavable N-terminal mitochondrial targeting sequences of cytochrome P450Scc and cytochrome P450 sterol 27-hydroxylase revealed that N-terminus domain of α-syn, which is rich in positively charged residues, mirrors the physico-chemical properties of mitochondrial targeting sequences and can adopt an α-helical conformation that can drive the anchoring of the protein to mitochondrial membranes (Ulmer et al., 2005; Devi and Anandatheerthavarada, 2010). The first N-terminal 32 amino acids have been shown to be fundamental for mitochondrial localization of the protein, (Devi et al., 2008) and, in particular, the lack of the first 11 N-terminal amino acids almost completely suppressed the *in vitro* binding of exogenous α-syn to isolated mitochondria from human HEK293 cells (Robotta et al., 2014).

As a matter of fact, all the PD-related mutations occur within the α-syn N-terminal domain, raising the interesting possibility that, beside their effect on protein aggregation (Conway et al., 1998; Li et al., 2001; Choi et al., 2004; Greenbaum et al., 2005; Sahay et al., 2017), they could impact on α-syn association to intracellular membranes and thus on its subcellular localization.

Interestingly, some mutants showed a greater tendency to be imported into mitochondria as compared with their wt counterpart (Cole et al., 2008; Devi et al., 2008; Guardia-Laguarta et al., 2014; Pozo Devoto et al., 2017), highlighting a potential pathogenic mechanism of action.

At the functional level their mitochondrial import was associated with broad mitochondrial defects such as increased mitochondrial Ca^2+^ levels, nitric oxide and ROS formation, cytochrome c release and apoptosis (Parihar et al., 2008, 2009), impairments of selected mitochondrial respiratory chain complexes (Subramaniam et al., 2014) and increased mitochondrial clearance (Chinta et al., 2010).

In the next sections, we will discuss the molecular basis for this intimate and functionally relevant relationship of α-syn with mitochondria, α-syn peculiar sub-organelle localization as well as the specific partners and processes that it governs at submitochondrial level (**Table 1**).

### Alpha-Synuclein at the Outer Mitochondrial Membrane

This intrinsic ability of α-syn to bind lipids and thus membranes, especially those with negatively charged surfaces, raised the possibility of potential interactions with the mitochondrial membranes (Shvadchak et al., 2011). The lipid binding properties of α-syn have been extensively investigated (Rhoades et al., 2006) and several studies have demonstrated the ability of α-syn N-terminal domain to adopt an α-helix conformation upon exposure to lipid surfaces (Ulmer et al., 2005). Deletion and/or insertion of charged amino groups in the first 25 residues of α-syn N-terminal domain strongly affected the propensity to adopt an α-helical conformation and also altered the binding to membranes (Perrin et al., 2000; Vamvaca et al., 2009; Bartels et al., 2010). Thus, it is not surprising that the presence of mutations in this domain strongly affected this feature. However, the three most frequent mutations confer different behavior: the A30P perturbs the helical structure leading to a reduction in lipid affinity, the E46K mutation increases the affinity for lipids, while the A53T has no major effect (Perrin et al., 2000; Jo et al., 2002; Bussell and Eliezer, 2004; Perlmutter et al., 2009).

By confocal and immuno-gold electron microscopy techniques a fraction of cellular α-syn has been found to localize at the OMM in dopaminergic neurons (Li et al., 2007) and rat brain neurons (Zhang et al., 2008). High pressure freeze immuno-electron microscopy on SH-SY5Y cells overexpressing α-syn further revealed that the protein directly binds the outer membrane of mitochondria leading to a MFN2 and DRP1-independent mitochondrial fragmentation without affecting the mitochondrial membrane potential or the ATP levels (Kamp et al., 2010). The expression of pathologic A30P and A53T mutants retrieved similar results, despite of in vitro analysis has revealed, at least for the A30P, a reduction in lipid affinity, thus suggesting that the amount of overexpressed protein could also play a major role in membranes binding (Kamp et al., 2010). Accordingly, upon overexpression of wt or A53T and E46K α-syn mutants, but not under conditions of reduced α-syn levels (Nakamura et al., 2011) or upon overexpression of the A30P mutant, a phenotype of mitochondrial fragmentation was observed suggesting that the amount of α-syn bound to mitochondria membranes might play a pivotal role. In line with this possibility, recent findings have shown that the forced delivery of wt and A53T α-syn to the outer membrane of mitochondria caused a reduction in mitochondrial size, while the A30P mutant had no effect (Pozo Devoto et al., 2017).

Compelling evidence for a selective preference of α-syn for mitochondria came from FRET-based and *in vitro* studies demonstrating that it selectively binds to mitochondria independently of the mitochondrial membrane potential, suggesting that the lipid composition rather than the functional state of the organelle is involved in the binding (Nakamura et al., 2008). These studies indicated that cardiolipin, a phospholipid enriched in mitochondrial membranes, is strictly required for α-syn interaction (Ghio et al., 2016).

Additionally, by immune-electron microscopy on HEK293 cells stably expressing α-syn it has been observed that under condition of low intracellular pH α-syn translocates to the OMM, but not within the mitochondrial matrix or the intermembrane space (Cole et al., 2008). Thus, it is tempting to speculate that the mitochondrial targeting of α-syn could be enhanced by cellular stress conditions. Whether the association of α-syn with mitochondria occurs directly with the membranes or is mediated by other proteins is still unclear. Sodium carbonate and proteinase K treatment on isolated mitochondria does not interfere with its association (Cole et al., 2008; Parihar et al., 2008). On the other hand, *in vitro* pull down experiments of mitochondrial extracts with a peptide composed of the last C-terminal 40 amino acids of α-syn retrieved TOM22, TOM40, VDAC 1-2-3 and Samm50 as binding partners (**Figure 2**) and, interestingly, S129 phosphorylation drastically reduced α-syn association with TOM40 and Samm50 (McFarland et al., 2008). Beside a series of α-syn induced mitochondrial alterations (such as increased ROS production and oxidative stress, alteration in complex I and deletions of mtDNA), α-syn accumulation was also accompanied by a decrease in TOM40 expression (Bender et al., 2013). More recently, α-syn binding to TOM20, but not to TOM40, TOM22 or Tim23, has been reported to impair the TOM20/TOM22 assembly, affecting the import of the complex I subunit Ndufs3 and leading to reduced respiration and increased ROS production. Intriguingly, the oligomeric dopamine-modified and the phosphomimic S129E mutant of α-syn, but not the monomeric or the nitrated and fibrillary forms, were reported to impair protein import, suggesting that the trimeric/tetrameric structure may play a role in mediating mitochondrial toxicity (Di Maio et al., 2016). As anticipated above, VDAC is also an important and recurrent α-syn binding partner: co-immunoprecipitation analysis revealed an association in the brainstem, striatum, and cerebral cortex of Thy1-A53T human α-syn transgenic mice (Martin et al., 2014) and *in vitro* studies revealed that recombinant monomeric α-syn at nanomolar concentration was sufficient to reversibly block VDAC1 channel activity in planar lipid bilayer, (Rostovtseva et al., 2015). Interestingly, nigral neurons from brains of PD patients containing α-syn positive inclusions also showed reduced VDAC1 immunoreactivity as compared with those displaying soluble or absent α-syn (Chu et al., 2014), suggesting a strong correlation between α-syn and VDAC in the pathogenesis of PD.

Thus, although α-syn possess the ability to bind cellular membranes directly, with an intrinsic preference for those mirroring the composition of the mitochondrial ones, protein-protein interactions with the components of the mitochondrial import machinery at the OMM have been consistently reported. Albeit a specific molecular mechanism for this selectivity toward the mitochondrial protein import machinery, in particular TOM20 and TOM40, is still missing, it might represent one of the first event in the neurodegenerative process.

### Alpha-Synuclein in the Inter-membrane Space (IMS)

Several studies have raised the possibility that a portion of the cellular α-syn can be found at the sub-mitochondrial level [inter membrane space (IMS), the inner membrane or the matrix], implying that a direct translocation from the cytosol across the OMM must also occur. As stated above, a number of OMM partners involved in this process has been found; however, how and why α-syn is imported into the mitochondria is still almost completely unexplored. *In vivo* and *in vitro* import of human α-syn into the mitochondria depends on the mitochondrial membrane potential and ATP levels and although anti-TOM40 antibodies have been reported to abolish α-syn mitochondrial localization (Devi et al., 2008), direct translocation through VDAC reconstituted into planar lipid bilayer has also been demonstrated (Rostovtseva et al., 2015; Hoogerheide et al., 2017), raising questions on whether there is a selective engagement on one of the two pathways or both components can be equally used based on the cell needs (Neupert and Herrmann, 2007). Understanding if the occurrence of the pathogenic mutations, the post-translational modifications or the aggregation state of the protein drive it to a specific pathway may be useful for a better understanding of α-syn physio/pathological behavior inside mitochondria as well as for the development of pharmacological approach and deserves further investigations.

**FIGURE 3:**
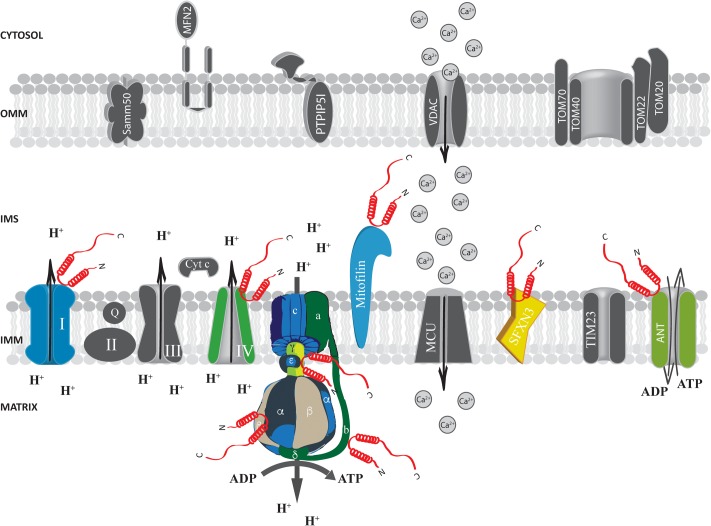
Alpha-syn at the inter membrane space (IMS), inner mitochondrial membrane (IMM) and mitochondrial matrix. Picture shows α-syn binding partners at the IMS, IMM and mitochondrial matrix. The protein’s interactors are highlighted in colors. Alpha-syn is shown in red.

At the IMS α-syn has been shown to interact with a portion of VDAC1 facing this compartment in the substantia nigra of α-syn overexpressing rats and in dopaminergic MN9D cells (Lu et al., 2013) and with the adenine nucleotide translocase (ANT) (**Figure 3**), one of the most abundant proteins of the inner mitochondrial membrane (IMM; Liu and Chen, 2013) involved in the exchange of ADP/ATP between the matrix and the IMS (Ryan et al., 1999; Halestrap and Brenner, 2003). Interestingly, alterations of mitochondrial membrane potential, increased ROS production and mitochondrial vacuolation, swallowing and loss of cristae observed upon α-syn overexpression are partially reverted by the incubation with the ANT inhibitor bongkrekic acid (BKA), which was shown to reduce the interaction between α-syn and both ANT and VDAC, suggesting that this interaction could be the key for α-syn detrimental activity (Shen et al., 2014). Interestingly, these effects could not be recapitulated by overexpression of a truncated form of α-syn lacking its N-terminus (aminoacids 1–60) or mutated in two key residues important for the targeting (the V63 and N65 residues), indicating that the N-terminus domain is critical for α-syn-induced cytotoxicity, probably affecting its mitochondrial localization (Shen et al., 2014; Zhang et al., 2016). Altogether, these findings suggest that α-syn translocation into the IMS of mitochondria might be a physiologically relevant under basal conditions and that increasing levels of α-syn also at this site could impact on the ability of cell to keep the ATP levels well balanced, contributing to the damage of dopaminergic neurons (Zhu et al., 2011).

### Alpha-Synuclein at the Inner Mitochondrial Membrane

Electron paramagnetic resonance (EPR) spectroscopy studies designed to analyze the interactions between α-syn and large unilamellar phospholipid vesicles mimicking either the inner or the OMM, indicated that the protein binds specifically to the IMM through its N-terminus (Robotta et al., 2014). In accordance, fluorescence anisotropy and high resolution nuclear magnetic resonance spectroscopy (NMR) studies confirmed that wt and A30P α-syn mutant could strongly interact with large unilamellar phospholipid vesicles mimicking the IMM but not with those mimicking the OMM. The differential affinity for the two membranes was related to the specific cardiolipin composition, which in the inner membrane is at least 40 times higher than in the outer membrane (de Kroon et al., 1997; Zigoneanu et al., 2012). This evidence was further confirmed in α-syn transgenic mice which showed a predominant α-syn localization at the IMM (Nakamura et al., 2011). Indeed, cardiolipin ablation from inner membrane-like vesicles or its saturation with cardiolipin-binding dye nonyl-acridine orange (NAO) strongly prevented α-syn binding (Cole et al., 2008; Zigoneanu et al., 2012).

Proteomics analysis identified a series of α-syn interactors at the level of the IMM, including mitofilin, a mitochondrial inner membrane protein important for the regulation of cristae morphology (John et al., 2005; McFarland et al., 2008). iTRAQ proteomics on synaptosomes from α-syn^+/+^ and α-syn^-/-^ mice also revealed that α-syn interacts with sideroflexin 3 (SFXN3), a putative iron transporter of the IMM (Fleming et al., 2001; Li et al., 2010) which is important for maintenance of the synaptic morphology and neuromuscular junctions (Amorim et al., 2017) and that has been found down-regulated in substantia nigra of PD-affected patients (Simunovic et al., 2009).

The mitochondrial solute carrier family 25 members (namely the 3, 11, 12 and 13) and the components of the electron transport chain have also been reported as binding partners of α-syn (McFarland et al., 2008). Accordingly, yeast two-hybrid and co-immunoprecipitation assay confirmed a specific interaction of α-syn with complex IV of the mitochondrial transport chain (Elkon et al., 2002). Moreover, association of α-syn with complex I in PD brain has also been reported and found to induce a reduction in complex I activity and increased ROS production in a time and dose-dependent manner (Devi et al., 2008; Liu et al., 2009), suggesting that the progressive accumulation of α-syn at the IMM may impair mitochondrial functions and induce oxidative stress.

### Alpha-Synuclein in the Mitochondrial Matrix

To the best of our knowledge, the number of studies indicating a specific localization for α-syn within the mitochondrial matrix is limited and evidence for α-syn translocation through the IMM is missing. Moreover, *ex vivo* proximity ligation assays reveal no interactions between α-syn and Tim23, the major protein translocase of the inner membrane of mitochondria (Di Maio et al., 2016). Nevertheless, some clues suggest the possibility that a minor fraction of α-syn could be localized at the mitochondrial matrix (Devi et al., 2008; Zhang et al., 2008; Liu et al., 2009) and, intriguingly, proteomic analysis revealed α-syn interaction with the B, D and γ chain of the ATP synthase (McFarland et al., 2008).

Evidence obtained at functional level showing that α-syn absence impact essential mitochondrial function further support the possibility that α-syn may interact with ATP synthase. Indeed, primary neuron/glia co-cultures from cerebral cortex of α/β/γ-syn triple knock out (TKO) mice revealed reduced mitochondrial membrane potential, decreased ATP synthase activity and lower ATP levels (Ludtmann et al., 2016), importantly, exogenous addiction of monomeric α-syn, but not of the A30P mutant, to the neuron/glia co-cultures was able to fully revert the phenotypes observed in the TKO neurons by physically interacting with the α subunit of the ATP synthase (**Figure 3**), suggesting that the mitochondrial matrix-resident fraction of α-syn could play a pivotal role in regulating important mitochondrial activities.

This scenario brings to speculate that a pool of α-syn exerts a physiological role inside the mitochondrial matrix where it is able to increase ATP synthase activity through direct binding with its α subunit, thus ensuring mitochondrial health and proper ATP fueling for synaptic functions. Aggregation and/or mutations of the protein could thus result in exaggerated mitochondrial accumulations that may lead to loss of function and initiate the degenerative process in PD.

## Conclusion and Future Perspectives

Mitochondrial dysfunctions and α-syn misfolding/aggregation have both been extensively documented in the pathogenesis of PD (Exner et al., 2012; Bose and Beal, 2016). The possible interplay between the two phenomena is suggested by the evidence that increased levels and/or mutations of α-syn trigger mitochondrial alterations and that mitochondrial impairment causes α-syn accumulation and aggregation (Betarbet et al., 2006; George et al., 2010). Which of the two events comes first in the development of the neurodegenerative process is not yet clear (Zaltieri et al., 2015), but the strong association between α-syn and mitochondria is nowadays believed to play a pivotal role in the pathogenesis of PD. Although substantia nigra pars compacta dopaminergic neurons are among the first neurons to degenerate, additional neuronal populations are also affected in PD (Dauer and Przedborski, 2003; Surmeier et al., 2017; Zhai et al., 2018). Despite the mechanisms governing this selective vulnerability are poorly understood, increasing evidence suggests that α-syn-mediated alteration of mitochondria wellness might be particularly important in dopaminergic neurons from substantia nigra (Calì et al., 2014). Indeed, they show increased cytosolic dopamine, Ca^2+^ entry and mitochondrial oxidative stress that, in turn, induces accumulation of oxidized dopamine and mitochondrial dysfunction, leading to the onset of the PD-related motor symptoms (Burbulla et al., 2017; Lieberman et al., 2017).

Despite the absence of a canonical mitochondrial targeting signal in α-syn sequence, a growing body of literatures indicates that at least a portion of cellular α-syn not only is able to physically interact with mitochondrial membranes by lipids-mediated binding, but also to cross them, possibly through a translocation process mediated by TIM/TOM complex or VDAC protein, reaching the intermembrane space and/or the matrix (Devi and Anandatheerthavarada, 2010; Nakamura, 2013; Abramov et al., 2017; Pozo Devoto and Falzone, 2017). Interestingly, α-syn effects on mitochondria seem to interplay with other mitochondrial proteins/pathways known to be mutated/altered in PD, further confirming that mitochondrial α-syn can be a major player in the onset of the disease. In fact, it has been proposed that blockage of mitochondrial protein import through α-syn interaction with TOM20 may activate Pink1/Parkin mediated mitophagy, by promoting Pink1 accumulation at the OMM and consequent Parkin recruitment (Di Maio et al., 2016). In accordance, midbrain dopaminergic neurons overexpressing α-syn A53T display an increase in mitochondrial clearance (Chinta et al., 2010), and the overexpression of wt Pink1 or Parkin, but not their PD-associated mutants, is able to rescue the α-syn-induced impairment of mitochondrial morphology (Kamp et al., 2010).

Several aspects of α-syn/mitochondria interplay, however, need to be further elucidated. First, it is worth to investigate whether the different sub-organelle localizations of the protein account for peculiar physiological functions or represent the response to specific stimuli. It is not clear whether α-syn toxic species are delivered to the mitochondria as a pathological or a protective mechanism. In other words, it is still to be proven whether the portion of α-syn that localizes within mitochondria resides there to exert a physiologically relevant function or for other reasons, i.e., to be degraded. An elegant work has shown, in yeast and human cells, that protein aggregates enter the mitochondrial intermembrane space and matrix (through the mitochondrial import machinery) in order to be degraded (Ruan et al., 2017). The possibility that this process also favors the clearance of α-syn deserves further investigations.

In addition, the subtle equilibrium between physiological and pathological roles of α-syn and its propensity to accumulate within the cell in the course of the neurodegenerative process suggest that there might be a threshold of protein amount in the cytosol and mitochondria that discriminates between healthy and detrimental effects. In accordance, it has been shown that the mitochondrial distribution of the wt and A53T α-syn is significantly increased in cells overexpressing the proteins compared to controls (Parihar et al., 2008) and that their induction of mitochondrial fragmentation is directly related to their expression levels (Pozo Devoto et al., 2017). Moreover, only high expression of the A53T mutant in midbrain dopaminergic neurons of transgenic mice significantly increases the number of autophagic mitochondria (Chinta et al., 2010). Finally, we have previously demonstrated that increased endogenous wt α-syn content by valproic acid treatment and the TAT-mediated delivery of high doses of exogenous protein lead to a reduction in mitochondrial Ca^2+^ uptake, while low levels of the protein induce an increase in mitochondrial Ca^2+^ influx. This observation could explain the discrepancies observed on α-syn action at the MAMs, being its effects possibly related to different transfection methods that permit to reach different amount of overexpressed α-syn (Calì et al., 2012; Paillusson et al., 2017).

Differences in α-syn oligomerization states could further complicate the scenario. It is unclear which aggregation state of the protein forces its mitochondrial localization and its detrimental or positive effects on mitochondria. Nevertheless, western blot analysis on rat brain purified mitochondria incubated with aggregated or un-aggregated α-syn revealed that only the oligomeric form of the protein can associate with mitochondria, as confirmed by immuno-gold electron microscopy (Parihar et al., 2008, 2009). Concordantly, only soluble and prefibrillar α-syn oligomers, but not monomeric or fibrillar α-syn, are able to bind TOM20 and block mitochondrial protein import as well as to induce complex I dysfunction and mitochondrial membrane potential dissipation leading to the impairment of mitochondrial Ca^2+^ handling and enhanced cytochrome c release (Luth et al., 2014; Di Maio et al., 2016).

Finally, the impact of known pathogenic mutations on α-syn mitochondrial regulation is not completely understood. Beside their role on protein aggregation, it has been suggested that mutations are able to regulate α-syn-mitochondrial association, with the A30P mutant being particularly defective in mitochondrial membranes binding.

Albeit all this evidence clearly supports a pivotal role for α-syn in the alteration of mitochondrial functions leading to the neurodegenerative process, they also point out the requirement of additional efforts to dissect the intimate relationship between α-syn physio/pathology and mitochondrial dysfunctions, providing new elements for the complete understanding of neuronal degeneration in PD.

## Author Contributions

MV, DC, MB, and TC contributed to the design and writing of the manuscript.

## Conflict of Interest Statement

The authors declare that the research was conducted in the absence of any commercial or financial relationships that could be construed as a potential conflict of interest.

## Footnotes

1 http://parkinson.org/Understanding-Parkinsons/Causes-and-Statistics/Statistics

2 https://report.nih.gov/NIHfactsheets/ViewFactSheet.aspx?csid=109

3 https://www.proteinatlas.org/ENSG00000186868-MAPT/tissue
